# Desensitizing the Tooth Pulp by Way of the Dentinal Tubules

**Published:** 1905-08-15

**Authors:** J. Edw. Line

**Affiliations:** Rochester, N. Y.


### DESENSITIZING THE TOOTH PULP BY WAY OF THE DENTINAL TUBULES.

BY J. EDW. LINE, D.D.S., ROCHESTER, N. Y.

The discovery by Spooner that arsenious acids would destroy the tooth-pulp was an epoch-making bit of work in the history of dentistry, and was quickly followed by the application of this agent as an obtundent of sensitive dentine, whether for that purpose merely, as we use silver nitrate to-day, or as a part of the preparation of cavities of decay for the further operation of filling. The later discovery that its action had no limitation short of the dest-struction of the pulp led forthwith to its discontinuance, except as it is used at the present time and for the same purpose. And it may be of interest to note that the man chiefly responsible for its widespread use as an obtundent was among the first to cry out against further misuse of an otherwise invaluable adjunct to the dental armamentarium. He frankly admitted his error, shouldered his full share of the responsibility, which was great, and used every means within his power, whether of tongue or pen (for he spoke and wrote much and often) to stop those given to the practice and dissuate those quite ready to begin.

### GOOD AT TIMES.

Medicaments that merely anesthetize the terminal ends of the dental fibrils have long played an important part in cavity preparation, and even to-day there are men who pin their faith to and cinch their practice by using chloroform, ether, or one or more of the essential oils, singly or combined, as obtundents of sensitive dentine—and with good reason. Medicaments that destroy, but only very superficially, still have a place in our cabinets: chloride of zinc is, perhaps, the best representative and most generally used. Medicaments that dessicate—that dry—that rob the fibrils of their water—have been and are now occasionly made use of to good advantage. Alcohol of high grade and glycerine in its purest form rate high because of their exceptional greed for water.

Heat by means of the directly-applied hot burnisher; a jet of air; hot solutions unaided, or solutions driven home by the heated burnisher or heated air, add materially to the list.

The application of cold, or rather the abstraction of heat, by means of ether or rhigolene spray held sway for a spell, only to be dropped because of the small margin for choice between suffering of the disease and that caused in the attempt at cure.

### COCAINE.

To-day, while these have not been wholly displaced, all resort to that best of all-round local anesthetics (analgesis), cocain, or combinations of which it is of necessity the essential ingredient. Applied in aqueous solution it has some effect both on dentine and on the pulp; aided by pressure, continuous or intermittent, as in percussion, it goes to the depths. The same may be truthfully asserted of cocain and its combinations, only to an immeasurably greater degree when influenced or re-inforced by the electric current.

Cocain has held its place from the first, the medicament seemingly the thing most desired and needed; but methods and appliances have only recently extended its field of usefulness and made its effects more certain and speedy of attainment.

### BY WAY OF THE NOSE.

A method at once unique, and by those who have resorted to it very effective, is that first suggested by Le-Crone as possibly of value to the dentist, and advocated in certain cases by Low. It calls for the introduction into the nose anteriorly of a plegget of cotton saturated with a solution whose chief ingredients are cocain and adrenalin. This combination affects the nerve supply of the anterior teeth, sometimes the first bicuspids, more rarely the second, and, let us remember, not through the tubules of dentine, but through the nerves to the apical foramina and possibly through those of the substance of the pulp itself. It seems not to have become popular, and the chief reason for this may be charged to the necessity on the part of the patient of having with him for the remaining twenty-four hours a pocket mirror to re-assure himself from time to time that his nose and upper lip are still on.

### HIGH PRESSURE METHODS.

But with some all of these methods have become obsolete, and resort is had to the method that involves the use of a powerful syringe for forcing cocaine or its equivalent into and through the dentinal tubules. Like syringes generally those that apply here have, as an essential part of their make-up, a piston or plunger operated by direct pressure, as in the U. S.; the screw, as in the Sapp; the lever, as in the Meyers and the Wilcox.

Whether for the work of painless cavity preparation, or the removal of the pulp, the nozzle is implanted in dentine. To this end a pit must be made in the dental tissue, which is easily and quickly done in a cavity of decay, or on eroded or abraded surfaces from which the enamel has been removed. Otherwise the enamel must be perforated, and the beginning of this canal counter sunk, to provide for accidental lateral movement on the part of the rigid nozzle—a valuable bit of practice brought it to the attention of the profession early in the history of the method. Without this provision flaking or even deep fracture of the enamel may occur, to say nothing of the disagreeable wrenching of the tooth because of the fixing of the appliance when the nozzle is inserted into a deep pit. The whole instrument becomes for the time a powerful and correspondingly dangerous lever.

With these preparations and precautions the work consists in forcing into and through the dentinal tubules as much as possible of the contents of the syringe.

### THE DEPTH OF THE PIT.

It becomes a question of some interest as to the depth to which the pit should be made. In a cavity of decay it would seem that the deepest part, giving as it does the nearest approach to the pulp, would afford the best, because the shortest, route to that organ. But in superficial decay covering much surface and calling for extensive cutting, the number of tubuli included by the nozzle would leave a large zone unaffected. Here, two or more injections might be indulged in; but it would seem as if the same or a better result might be affected by limiting the depth of the pit to the granular layer—that but partially or loosely calcified layer that marks the dividing line between dentine and its covering tissues. Laboratory experiments show a wide spread of the injecting solution in this layer, and a greater number of tubules affected—a number proportionately numerous, to the affected part of the layer in question. Theoretically, at least, the greater the number of tubules carrying the solution pulp-wards the greater the surface desensitized. This inferential advantage is offset somewhat by the fact that, while but few tubules are included in the nozzle of the syringe, the solution driven through them to the pulp is distributed over a considerable surface of the pulp itself, anesthetizing more or less completely the whole district supplied by fibrils radiating from the affected pulp surface.

### SUCCESS AND FAILURE.

The inventor of one of the instruments in question reported some time since over one hundred cases without a single failure. These included cavity preparation, pulp extirpation and decrowning. And yet some of the last named might not have given any pain if this or any other means of desensitizing had not been employed. To illustrate: Having two central incisors to crown we notched them labio-ligually with a suitable wheel, thinking, meanwhile, in case they proved sensitive to resort to the desensitizing method under consideration. As there was no suggestion of pain on the part of the patient—a lady of about twenty—we clipped them both with a forcep and, cautiously inserting a broach into the pulp cavity of one, making it hug the wall, passed it to the end of the root, gave it a turn, and then laid the broach with its pulp on the table. In an equally sly way we repeated the operation on the other pulp with a like result. While engaged for the moment in winding cotton on a pair of dressing broaches the patient, examining the display on the table, asked, "Are those the nerves?" "Yes." "And everybody told me it would hurt!" This is an exceptional case, and yet, had we injected the teeth, two grand successes would have been duly recorded.

Notwithstanding the great success that comes to some men, failure, partial or complete, now and then comes to others. Wishing to decrown a lateral incisor we drilled a pit in the enamel of the mesial surface about one-third the length of the crown from the incisive edge and, to make doubly sure, another in a shallow, cervical cavity on the same surface. Decrowning and pulp extirpation were accompanied by pain the quality of which was beyond compare, the quantity sufficient to entangle him for a time in words sometimes used in prayer. Why this unlooked for result?

This patient was forty-five to fifty years of age, his teeth of exceptional quality, all of them bearing the marks of vigorous use. His age, coupled with the fact that the tooth showed much wear across the incisive edge, suggested calcified dentin, or rather filled-in or obliterated tubules, of course impervious to the solution. Opposite the cavity of decay we found a deposit on the pulp-cavity wall; and when it is called to mind that the tubules in these deposits are offset rather than continuous with those normally developed dentine, we have good and sufficient reasons for the failure. The better plan in these cases would be to open through a normal district, avoiding all places suggesting abnormal calcification and secondary deposits. This and similar cases tell us unmistakeably that attempts at injection of completely calcified dentine, or where there are secondary deposits, should not be made.

This kind of dentine, or dentine in this condition, is found in the aged and can be accounted for by age alone. It is also found in teeth long subject to abrasion. It is found in teeth affected by erosion and especially in those in which the erosive action intermits, or has received a permanent check. It is found in cavities of slow growth ; between the ebony-color line and the pulp-chamber. Failure should also follow in cases of secondary deposits on the wall of the pulp-cavity opposite the cavity of decay, as in the case of the elderly patient just mentioned. It should follow in case of pulp-stone that becomes adherent to the pulp-cavity wall.

### EXPOSED PULPS AND LARGE FORMANIA.

Success in this operation is modified or unattained in two other classes of cases. The first, that of exposed pulps; the greater the exposure the more likely the failure. Here the solution passes to the pulp and, following the line of least resistance, flows to and through the opening of the pulp-cavity into the cavity of decay. It is not held in place long enough to penetrate the tissues—not long enough in extensive exposures to appreciably affect the most superficial portions. Many of this class may be made successes provided the exposure is simple, easily reached and covered with cement before the injection is begun. In the second class of these cases we have similar conditions, but due to widely different causes. In the large apical foramina of undeveloped teeth of children, and the foramina of the adult when these are naturally but abnormally large, or made so from whatever cause, it is impossible to force the solution in other direction than the apex, where it must lay until disposed of by the comparatively slow-acting circulatory fluids.

### WHITHER THE SOLUTION.

What becomes of the cocain is already a matter of investigation in other quarters. In the case of exposed pulps it escapes largely into the cavity of decay. In cases of abnormally large apical foramina it trends toward the apical space, and beyond, to be gathered up as already stated. But without these outlets? Here it would seem, and laboratory experiments point that way, that it first gathers as a droplet, a sort of bleb, between the cavity wall and the pulp, eventually undergoing absorption by the vascular supply, and through this is eliminated from the plup and finally from the system itself. That it does reach the very interior of the pulp is proved, first, by the absence of pain when a broach is forced into a recently injected and decrowned tooth; second, by the finding of cocain in the substance of the pulp itself.

This latter calls for a tooth recently injected by way of the dentine, preferably in the mouth. The tooth is then extracted, notched as for decrowning, and placed near enough to the pipes of a cold storage box, or in ice and salt freezing mixture, to freeze or, at least, thoroughly chill it. Later the tooth is broken, the pulp showing a clean fracture, the whole quickly washed in running water. and a speck of the heart of the pulp placed in a drop of water on a slide and transferred to a bell-jar containing a bit of wet blotting paper. Under these circumstances evaporation of the water on the slide occurs slowly, with the result that we have cocain crystals in perfection. That they are such is further proved by the polariscope.

### TORN AND DISPLACED.

Another question that suggests itself is—What effect has this means of desensitizing the pulp on the soft structures of that organ, and especially the soft structures of the dentine? In laboratory experiments there is every appearance of the fibrills having been torn from their moorings and therefore to that extent the pulp itself is lifted from its anchorages in the pulp-cavity wall. Whether harm comes of this to teeth treated for filling is still an open question; but it certainly is worth considering in young teeth where the apical foramina are necessarily large, the soft tissues in such openings offering but little resistance to the force exerted in driving the solution home. In other cases the detachment and breaking up of the soft tissues is probably of no consequence.

MECHANICS OR CHEMISTRY?

To what is this desensitized condition of the pulp and its ramifications due? To the solvent, or to the cocain, or to both? The question comes because of the fact that similar—not the same—results have been obtained with water, and with water holding in solution substances that have no anesthetic effect whatever. Distilled water and water containing sodium chloride have been used in mucus serus and cutaneous surface work and no pain experienced the mere gorging of the vessels and adjacent tissues seeming to answer the purpose for the time being, much as a string tied about the finger acts by cutting down the local circulation. The desensitizing here is evidently due to the mechanics involved, rather than to the chemistry of the injected fluid. But whatever the effects of the solvent, cocain or its equivalent is an essential in this kind of work, and, except for mere experimental purposes for comparison, the matter would be hardly worth more than mention. It merely shows what can be done.

METHOD ANTICIPATED.

Finally, let all who have use cocain on a bur in excavating for filling, or for encroachment on the pulp with reference to extirpation, or on a wheel as a lubricant in cutting down stumps for crowning, take credit to themselves that they have anticipated the instruments now used for desensitizing purposes. To cut the ends of the tubules with a bur or grind with a wheel wet with cocain is to rub, squeeze, crowd, force into the tubules and their contents the desensitizing solution, and, as we lessen the distance between the instrument and the pulp cavity, into the pulp itself, so that when exposed the cornual end is partially and sometimes wholly desensitized. The conditions of success are that the surface shall be kept wet, that the bur or wheel shall be fine, and that its progress shall be comparatively slow, now and then to the turning of it backwards.—*Office and Laboratory.*

